# Associations between social alienation, psychological resilience, perceived intergenerational communication, and perceived care-related autonomy among rural older adults in China

**DOI:** 10.3389/fpubh.2026.1748217

**Published:** 2026-04-07

**Authors:** Qi Sun, Min Wang, Suning Shi, Shixue Zhou, Zhaoquan Jiang

**Affiliations:** 1 The First Hospital of Jinzhou Medical University, Jinzhou, Liaoning, China; 2 Nursing Department, The 960th Hospital of the Joint Logistics Support Force of the People’s Liberation Army, Jinan, Shandong, China; 3 School of Nursing, Jinzhou Medical University, Jinzhou, Liaoning, China

**Keywords:** perceived care-related autonomy, perceived intergenerational communication, psychological resilience, rural seniors, social alienation

## Abstract

**Background:**

The intricate nexus between social alienation and perceived care-related autonomy, particularly in rural contexts, remains underexplored. This study aims to dissect this relationship by focusing on the mediating influence of psychological resilience and the moderating impact of perceived intergenerational communication among rural older adults populations.

**Methods:**

This investigation employed a multistage stratified sampling technique to gather data from 1,025 rural seniors (aged 60 and above) in Liaoning Province, China, over the period from February 17, 2021, to April 20, 2023. The survey instruments encompassed the Generalized Social Alienation Scale, Psychological Resilience Scale, Perceived Intergenerational Communication Scale, and Perceived Care-related Autonomy Scale. Descriptive statistics were utilized for sample characterization. Linear regression analyses were conducted to scrutinize the link between intergenerational support from offspring and the seniors’ perceived care-related autonomy. Statistical analyses were executed using SPSS (version 26) and PROCESS (version 4.1).

**Results:**

(1) Social alienation was negatively correlated with psychological resilience and perceived care-related autonomy and perceived intergenerational communication. Psychological resilience was positively associated with perceived intergenerational communication and perceived care-related autonomy. Perceived intergenerational communication was positively correlated with perceived care-related autonomy. (2) The mediation analysis showed that social alienation had a direct associations on the perceived care-related autonomy, and psychological resilience partially mediated the relationship; Perceived intergenerational communication moderates on the path to “social alienation → psychological resilience → perceived care-related autonomy”; Perceived intergenerational communication moderates the latter part of “social alienation → psychological resilience → perceived care-related autonomy.”

**Limitations:**

(1) This research is a one-time observation study; (2) because of data constraints, other influencing factors may have been overlooked.

**Conclusion:**

This study delineates that under the mediating influence of psychological resilience, social alienation potentially diminishes perceived care-related autonomy. Additionally, the perception of intergenerational communication plays a dual moderating role: it not only influences the dynamic between social alienation and psychological resilience but also affects the correlation between social alienation and perceived care-related autonomy. These insights contribute significantly to the nuanced understanding of the interplay between social alienation and perceived care-related autonomy among the older adults.

## 1 Introduction

In recent years, China’s rapid socio-economic development and accelerated urbanization have led to a steady increase in the number of rural empty-nest older adults—defined as those aged 65 years or older living alone or only with a spouse due to their adult children migrating to urban areas for work (1, 2). This demographic shift not only intensifies the caregiving burden on families and society but also profoundly undermines the physical and mental health of these individuals. Statistics indicate that approximately 60% of rural older adults in China are empty-nesters, facing heightened risks of social and geographic isolation compared to their urban counterparts (2). Globally, similar challenges persist; for instance, in 2018, 20% of the European Union’s 101 million older adults (aged 65+) resided in predominantly rural areas, where geographic and social barriers exacerbate health and well-being deficits (3–5). Rural empty-nest older adults in China face additional vulnerabilities: about 50% experience some form of disability (versus a national average of 34%) (6, 7), limited formal care services impose heavier caregiving demands (8), and their incomes are roughly 40% lower than urban peers, constraining older adult care resources (9).

### 1.1 Theoretical basis and construct motivation

To address the challenges facing the growing population of rural empty-nesters, this study focuses on four psychosocial constructs selected for their specific relevance to the “aging in place” dilemma. The selection of these variables is motivated by the urgent need to understand how social disconnection impacts the functional independence of older adults and identifying protective factors that can sustain their ability to live alone.

### 1.2 Social alienation

In the context of rapid urbanization and the out-migration of adult children, social alienation has emerged as a critical risk factor for rural older adults. It is defined as a state of disconnection from one’s social network, characterized by loneliness, a lack of social support, and a diminished sense of belonging (4, 5). Unlike “social isolation,” which measures objective network size, social alienation captures the subjective, psychological experience of estrangement (10). Investigating this construct is imperative because it represents the primary psychological cost of the empty-nest phenomenon. Understanding the severity of alienation is the first step in determining how the erosion of traditional family structures impacts the mental landscape of the rural older adults.

### 1.3 Perceived care-related autonomy

While health outcomes are frequently studied, perceived care-related autonomy is arguably more critical for empty-nesters who must manage their lives without daily assistance. This construct refers to older adults’ self-perceived capacity to independently meet daily needs, manage emergencies, and maintain health without undue reliance on others (11). Rooted in Self-Determination Theory (12), it distinguishes itself from mere physical ability by emphasizing the psychological sense of agency. Understanding this construct is vital because it is the primary determinant of whether an older adult can successfully “age in place” or requires institutionalization. Therefore, preserving this autonomy is a key public health priority.

### 1.4 Psychological resilience

Defined as an individual’s adaptive capacity to recover from adversity through positive coping and resource utilization (13, 14), resilience is included here as a potential internal mechanism. It serves to explain why some alienated adults maintain their independence while others decline.

### 1.5 Perceived intergenerational communication

This construct encompasses the subjective perception of mutual understanding, emotional support, and quality interactions with younger family generations (e.g., adult children), facilitated by digital platforms or visits (15, 16). Its inclusion as a moderator is motivated by the specific context of the sample: for empty-nesters, the physical absence of children is the primary stressor. Therefore, the quality of communication serves as the most logical external resource to bridge this physical gap, potentially offsetting the functional deficits caused by alienation.

These constructs are integrated via Conservation of Resources (COR) theory (17), which posits that social alienation represents a depletion of psychosocial resources, subsequently impairing the resource of autonomy. Furthermore, Stress-Buffering Theory (18) is employed to explain how intergenerational communication can interrupt this depletion process.

### 1.6 Literature review and hypotheses development

#### 1.6.1 The association of social alienation on perceived care-related autonomy and the role of psychological resilience

Prior research has established a robust negative relationship between social alienation and functional outcomes. However, the specific pathway from social alienation to perceived care-related autonomy requires deeper investigation. Social alienation fosters a sense of helplessness and reduces self-efficacy, which directly undermines an older adult’s confidence in their ability to manage health and daily emergencies (4, 5, 19). This link is particularly salient in Chinese rural contexts (6, 20), where cultural expectations of filial piety make the experience of social alienation more psychologically damaging (1, 2).

However, this relationship is likely not direct. We posit that psychological resilience acts as the crucial mechanism (mediator). According to COR theory, alienation depletes the emotional energy required to maintain resilience. When resilience—the capacity to adapt and cope—is eroded, the older adult loses the psychological “fuel” necessary to maintain a sense of autonomy (13, 14, 21, 22). While Jeste et al. (21) found that resilient adults maintain autonomy despite isolation, this mediation pathway has rarely been tested specifically among rural empty-nesters (23).

*Hypothesis 1 (H1)*: Psychological resilience mediates the negative relationship between social alienation and perceived care-related autonomy among rural empty-nest older adults.

#### 1.6.2 The moderating role of perceived intergenerational communication

A critical gap in the literature is the lack of understanding regarding conditional factors that might protect autonomy in the face of alienation. We introduce perceived intergenerational communication as a dual-stage moderator based on its relevance to the specific needs of this population.

First, regarding the social alienation → psychological resilience path: High-quality communication acts as a primary buffer. Even when an older adult feels alienated from their immediate community, strong communicative links with distant children provide emotional validation and a sense of “remote” support (15, 16, 24). This influx of social resources prevents alienation from depleting the individual’s internal resilience.

*Hypothesis 2 (H2)*: Perceived intergenerational communication moderates the negative relationship between social alienation and psychological resilience, such that the relationship is weaker at higher levels of intergenerational communication.

Second, and crucially, regarding the alienation → autonomy path: Communication is directly relevant to care-related autonomy. Regular, high-quality communication provides informational support (e.g., health advice, safety checks) and emotional reassurance, which directly bolsters the older adult’s confidence in their ability to live independently (15, 25). By maintaining a “psychological presence” of the caregiver, communication empowers the empty-nester to feel autonomous rather than abandoned, thereby neutralizing the paralyzing effect of alienation on their perceived capacity for self-care (26). This dual-moderation approach (consistent with Hayes’ PROCESS Model 7 (27)) offers a nuanced understanding of how family dynamics can preserve independence despite physical separation.

*Hypothesis 3 (H3)*: Perceived intergenerational communication moderates the negative relationship between social alienation and perceived care-related autonomy, such that the relationship is weaker at higher levels of intergenerational communication.

**Figure 1:**
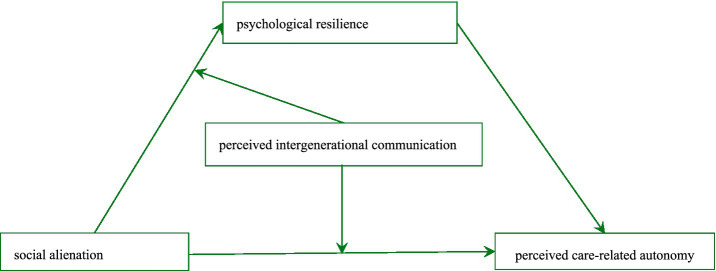
Proposed theoretical framework.

Figure 1 illustrates this moderated mediation model. By systematically mapping these relationships, this study moves beyond simple associations to explain how (via resilience) and under what conditions (via communication) rural empty-nesters can maintain the autonomy necessary for aging in place.

## 2 Materials and methods

### 2.1 Participants

#### 2.1.1 Data collection methodology

A multi-stage, stratified sampling approach was employed from February 17, 2021, to April 20, 2023, targeting rural seniors in 10 townships across three counties (Chaoyang, Beipiao, and Jianping) in Chaoyang City, Liaoning Province, China. The selection process involved a random number table for unbiased sampling. Initially, the eight counties in Chaoyang City were numbered and three counties were randomly chosen. Subsequently, townships within these counties—25 in Chaoyang County, 27 in Beipiao County, and 24 in Jianping County—were numbered and three from each were selected using the same random method.

#### 2.1.2 Participant recruitment and inclusion

The research participants, rural seniors, were identified in collaboration with local civil affairs departments, adhering to inclusion criteria: willingness to participate, clear cognitive and communicative abilities, no history of neurological or psychiatric disorders, and registered as rural, permanent residents aged 60 or older. A total of 1,115 questionnaires were distributed, with 1,025 valid responses obtained, yielding a 91.92% response rate.

#### 2.1.3 Data collection procedures

Given the COVID-19 pandemic, data were collected via a mixed-mode approach combining face-to-face structured interviews (primary for low-literacy participants) and online self-completion (via WeChat/secure platform for tech-savvy respondents, with assistant guidance if needed). Trained research assistants administered face-to-face sessions (reading items aloud/recording verbatim) and facilitated online distribution/submission, ensuring accessibility and compliance with local health protocols (e.g., masking/distancing). A total of 1,115 questionnaires were distributed. Of these, 1,025 valid responses were collected, while 90 questionnaires were classified as invalid due to either exceeding 20% missing data or containing inconsistencies. This resulted in a valid response rate of 91.92%.

The current research received ethical approval from the Medical Ethics Committee of Jinzhou Medical University, evidenced by approval number JZMULL2022017. Informed consent in written form was secured from all participants involved in the study. Furthermore, all procedures undertaken in this study conformed to the principles outlined in the Declaration of Helsinki.

### 2.2 Measurement

#### 2.2.1 General Social Alienation Scale

##### 2.2.1.1 Assessment of social alienation

The Generalized Social Alienation Scale, developed by Wu et al. (10), was employed to evaluate the participants’ perceptions of alienation and hesitancy in engaging in various activities. This study utilized the 15-item version of the Social Alienation, which has been established as a tool with substantial validity and reliability, demonstrated by a Cronbach’s *α* of 0.77. The scale is divided into four distinct dimensions: feelings of social alienation, self-alienation, sense of meaninglessness, and perception of powerlessness (12). Responses were recorded using a four-point Likert scale ranging from 1 (strongly disagree) to 4 (strongly agree). The overall scoring spectrum spans from 15 to 60, where elevated scores signify greater levels of social alienation. Sample items include “Sometimes I feel that the people I know are not very friendly” for social alienation and “Most of the things I do daily are very valuable and meaningful to me” for meaninglessness. In the context of this study, the Cronbach’s *α* for the complete 15-item Social Alienation was 0.805, while the *α* values for the subscales varied from 0.614 for the powerlessness dimension to 0.772 for the self-alienation component. This limitation is acknowledged and discussed in the limitation section.

#### 2.2.2 Psychological Resilience Scale

##### 2.2.2.1 Measurement of psychological resilience

The study utilized the Psychological Resilience Scale, originally crafted by Wagnild and Young (17) and later translated into Chinese by Huang (18). This instrument encapsulates five facets of resilience: equanimity, perseverance, self-reliance, significance in life experiences, and serenity, encompassing a total of 25 items. The scoring for each item ranges from “1” (strongly disagree) to “7” (strongly agree), with the aggregate score extending from 25 to 175. Scores above 145 denote a high level of resilience, scores between 125 and 145 suggest a moderate level, and scores below 125 indicate a low level of resilience. Sample items include “I can get through difficult times because I’ve experienced difficulty before” for persistence and “When I make plans, I will follow through with them” for self-reliance. The overall reliability of the scale, as indicated by its Cronbach’s alpha, is 0.943. The alphas for the individual subscales vary between 0.797 and 0.913.

#### 2.2.3 Perceived care-related autonomy

##### 2.2.3.1 Evaluation of perceived care-related autonomy

The perceived care-related autonomy scale, designed by Pang (19), is grounded in Dorothea Orem’s self-care deficit theory (22) and integrates elements of the Active Aging Theory (23). This scale, specifically adapted for the Chinese older adults population, aims to assess their self-care capabilities. It encompasses three primary dimensions: financial self-sufficiency, proficiency in daily living activities, and health self-management, comprising a total of 45 items. Scoring for each item ranges from 1 (Not at all applicable) to 5 (Completely applicable), resulting in a total score spectrum of 45 to 225. Higher scores indicate a stronger perceived care-related autonomy among rural seniors. Sample items include “I have adapted to the current living environment (living pace, weather, etc.)” for daily living skills and “I can maintain good interpersonal relationships” for health self-maintenance. The overall reliability of the scale, as indicated by its Cronbach’s alpha, is 0.872. The alphas for the individual subscales vary between 0.781 and 0.905.

#### 2.2.4 Perceived intergenerational communication

The study utilized the Chinese version of the Perceived Intergenerational Communication Scale, translated by Ling et al. (27). Originally developed by Williams et al. (26) in 1997 based on Communication Accommodation Theory, the perceived intergenerational communication was initially designed to compare the differences perceived by young people in intergenerational communication with older adults across Eastern and Western cultural contexts. Subsequently, the scale has been employed to assess differences in older adults’ communication perceptions, demonstrating good reliability and validity in various countries, including South Korea, Japan, and the Philippines (28–31). The Chinese version of the perceived intergenerational communication is a self-report instrument, comprising four dimensions with a total of 32 items. These dimensions include Maladaptation (13 items), Adaptation, Respect (9 items), and Assessment of Intergenerational Communication: The evaluation includes a dimension focusing on “Avoidance”, comprising four items. Responses are gauged using a 7-point Likert scale, where 1 signifies “strongly disagree” and 7 denotes “strongly agree”. The computation of average scores for each dimension is undertaken, with higher averages reflecting more favorable perceptions of intergenerational communication. The reliability of this scale is underscored by an overall Cronbach’s alpha of 0.902, and the subscales demonstrate high internal consistency with alphas ranging from 0.829 to 0.966.

### 2.3 Covariates

Covariates in the Study: This research incorporated several covariates: age, gender, education level, monthly income, marital status, and chronic disease prevalence. Age was segmented into three brackets: 60–70, 70–80, and 80 years or older. Gender categorization included male and female. Education levels were classified as elementary or below, middle school, and high school or above. Monthly income levels were divided into <1,000 CNY, 1,000–2,000 CNY, and >2,000 CNY. Marital status was bifurcated into married and widow/widower. Lastly, the count of chronic diseases was classified into four groups: none, one, two, and three or more diseases.

### 2.4 Statistical analysis

Statistical Analysis Procedure: Initially, SPSS 26.0 was utilized for conducting descriptive statistics and correlation tests. Subsequently, the indirect influence of psychological resilience on the link between social alienation and perceived care-related autonomy was examined using Hayes’ PROCESS macro (Model 4) (32). For estimating the standard error of this indirect associations, a bias-corrected bootstrapping method with 5,000 samples was employed. Furthermore, the moderated mediation analysis (PROCESS Model 8) was applied to explore if the perception of intergenerational communication moderated both direct and indirect pathways. Prior to analysis, all variables were mean-centered. The final step involved calculating conditional indirect associations to assess how varying levels of perceived intergenerational communication impacted the indirect associations of psychological resilience. Demographic variables were consistently controlled throughout the statistical analyses.

## 3 Results

### 3.1 Characteristics of participants

Study participants and demographic analysis: The participant pool consisted of 1,025 rural seniors. Table 1 presents the demographic composition and univariate analysis results for associations between perceived care-related autonomy and demographic characteristics. The gender distribution was 459 males (44.78%) and 566 females (55.22%). Participants’ ages ranged from 61 to 82 years, with a mean age of 70.34 years (SD = 8.86). The overall mean perceived care-related autonomy score was 145.92 (SD = 21.72), suggesting moderate levels relative to the scale’s potential range (typically 0–200 for similar autonomy scales). Univariate analyses revealed significant associations between perceived care-related autonomy and demographic factors (e.g., higher perceived care-related autonomy scores among younger, more educated, higher-income, married participants, and those with fewer chronic diseases; all *p* < 0.05; detailed in Table 1). These descriptive patterns provide context for subsequent multivariate associations.

**Table 1:** One-way analysis of the perceived care-related autonomy among rural seniors with different characteristics (*N* = 1,025).

Variables	Group	*N*	Mean ± SD	*F*/*t*	*p*
Gender	Male	459	147.64 ± 22.62	4.040	0.023
	Female	566	144.53 ± 20.88		
Age	60~	444	152.86 ± 22.25	57.094	0.000
	70~	349	144.12 ± 16.01		
	80~85	232	135.33 ± 23.34		
Education level	Elementary school or lower	313	140.75 ± 26.15	20.884	0.000
	Middle school or less	565	146.59 ± 17.48		
	High school or higher	147	154.32 ± 23.19		
Monthly income	Less than 1,000 CNY	371	142.48 ± 25.77	8.941	0.000
	1,000~2,000 CNY	519	147.11 ± 18.09		
	Above 2,000 CNY	135	150.78 ± 21.07		
Marital status	Married	647	148.92 ± 19.86	7.251	0.000
	Widow, widower	378	140.78 ± 23.74		
Types of chronic diseases	0	213	152.38 ± 23.19	15.069	0.000
	1	346	146.95 ± 18.56		
	2	309	144.48 ± 18.17		
	Greater than or equal to 3	157	137.70 ± 28.64		

### 3.2 Correlation analysis of key variables

Pearson correlations showed significant negative associations between social alienation and perceived care-related autonomy (*r* = −0.533, *p* < 0.01), psychological resilience (*r* = −0.566, *p* < 0.01), and perceived intergenerational communication (*r* = −0.160, *p* < 0.01). Positive associations were observed between psychological resilience and both perceived care-related autonomy (r = 0.818, p < 0.01) and perceived intergenerational communication (*r* = 0.570, *p* < 0.01), as well as between perceived intergenerational communication and perceived care-related autonomy(*r* = 0.589, *p* < 0.01; see Table 2).

**Table 2:** Descriptive statistics and correlation analysis of social alienation, psychological resilience, perceived intergenerational communication and perceived care-related autonomy.

Variables	Mean ± SD	1	2	3	4
Social alienation	42.30 ± 14.05	1			
Psychological resilience	127.71 ± 24.57	−0.566^∗∗^	1		
Perceived intergenerational communication	162.87 ± 28.52	−0.160^∗∗^	0.570^∗∗^	1	
Perceived care-related autonomy	145.92 ± 21.72	−0.533^∗∗^	0.818^∗∗^	0.589^∗∗^	1

^∗∗^represents *p* < 0.01.

### 3.3 Mediation analysis

#### 3.3.1 Testing of hypothesis 1

Controlling for demographics (gender, age, education, income, marital status, chronic diseases), Model 4 of Hayes’ PROCESS 4.1 revealed an indirect association between social alienation and perceived care-related autonomy via psychological resilience [*β* = −0.597, SE = 0.041, 95% CI = (−0.675, −0.519)]. A direct association also existed [*β* = −0.161, SE = 0.036, 95% CI = (−0.232, −0.090)], indicating psychological resilience partially accounted for the social alienation–perceived care-related autonomy association (indirect: 78.76%; direct: 21.24% of total effect; Tables 3, 4).

**Table 3:** Associations in the mediation model (psychological resilience as mediator).

Variables	Psychological resilience				Perceived care-related autonomy			
	*β*	SE	*t*	95% CI	*β*	SE	*t*	95% CI
Gender	0.721	1.487	0.485	−2.197, 3.641	−1.193	0.916	−1.302	−2.990, 0.604
Age	−1.812	1.189	−1.523	−4.146, 0.522	−0.391	0.733	−0.534	−1.830, 1.047
Education level	1.909	1.761	1.083	−1.547, 5.365	0.796	1.085	0.734	−1.332, 2.926
Monthly income	1.065	1.648	0.646	−2.169, 4.299	−1.45	1.014	−1.429	−3.442, 0.540
Marital status	7.361	2.257	3.260^∗∗^	2.931, 11.791	−2.328	1.397	−1.666	−5.070, 0.412
Types of chronic diseases	−4.977	1.333	−3.732^∗∗^	−7.594, −2.360	3.124	0.826	3.779^∗∗^	1.502, 4.746
Social alienation	−0.878	0.052	−17.001^∗∗^	−0.979, −0.776	−0.161	0.036	−4.470^∗∗^	0.6428, 0 0.718
Psychological resilience					0.680	0.019	35.243^∗∗^	0.642, 0.718
*R* ^2^	0.343				0.682			
*F*	66.579				241.875			

^∗∗^represents *p* < 0.01.

**Table 4:** Results for associations of social alienation on perceived care-related autonomy with psychological resilience as a mediator.

Path	Associations value	Boot S. E.	Boot LLCI	Boot ULCI
Direct association	−0.161	0.036	−0.232	−0.090
Indirect association	−0.597	0.041	−0.675	−0.519
Total association	−0.758	0.047	−0.851	−0.665

### 3.4 The moderation analyses

#### 3.4.1 Evaluating hypotheses 2 and 3

The study employed Hayes’ PROCESS macro (Model 8) to examine moderated mediation associations. Two distinct models were analyzed: Model 1 evaluated the moderating association of perceived intergenerational communication on the association between social alienation and psychological resilience. Model 2 assessed the moderating impact of perceived intergenerational communication on the relationship between psychological resilience and perceived care-related autonomy.

**Figure 2:**
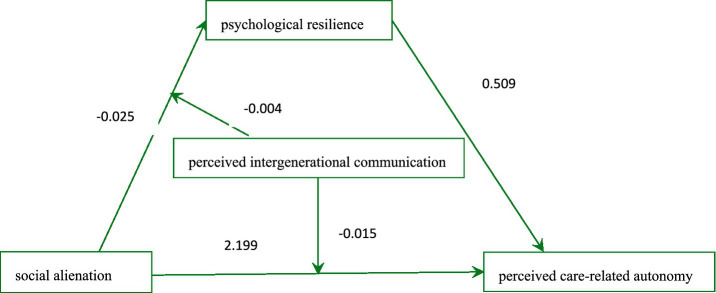
Model of moderated mediation analysis. The values shown are the standardized coefficients.

Findings, as depicted in Table 5, indicate that in Model 1, social alienation was significantly related to psychological resilience [*β* = −0.025, SE = 0.280, 95% CI = (−0.174, −0.011)], with perceived intergenerational communication acting as a moderator [*β* = −0.004, SE = 0.002, 95% CI = (−0.007, −0.001)]. In Model 2, a substantial association of social alienation on perceived care-related autonomy was observed [*β* = 2.199, SE = 0.191, 95% CI = (1.823, 2.572)], alongside a moderating association by perceived intergenerational communication [*β* = −0.015, SE = 0.001, 95% CI = (−0.017, −0.013)]. Consequently, hypotheses 2 and 3 received partial support. The finalized mediation model is illustrated in Figure 2.

**Table 5:** Outcomes from the analysis of the moderated mediation model.

Variables	Psychological resilience				Perceived care-related autonomy			
	*β*	SE	*t*	95% CI	*β*	SE	*t*	95% CI
Gender	1.392	1.179	1.181	−0.921, 3.706	−0.583	0.807	−0.723	−2.166, 0.999
Age	−0.569	0.945	−0.602	−2.423, 1.285	−0.466	0.646	−0.722	−1.734, 0.801
Education level	1.588	1.397	1.137	−1.153, 4.329	0.506	0.956	0.529	−1.369, 2.381
Monthly income	2.213	1.307	1.694	−0.351, 4.776	−0.799	0.894	−0.894	−2.554, 0.956
Marital status	2.277	1.807	1.260	−1.269, 5.822	−2.195	1.236	−1.775	−4.621, 0.231
Types of chronic diseases	−3.007	1.063	−2.829∗∗	−5.093, −0.921	2.520	0.729	3.455∗∗	1.089, 3.951
Social alienation	−0.025	0.280	−0.088∗∗	−0.174, −0.011	−2.199	0.191	11.485∗∗	−2.575, −1.823
Psychological resilience					0.509	0.021	23.696∗∗	0.467, 0.551
Perceived intergenerational communication	0.598	0.067	8.996∗∗	0.468, 0.729	0.752	0.047	15.925∗∗	0.660, 0.845
Social alienation × perceived intergenerational communication	−0.004	0.002	−2.483∗∗	−0.007, −0.001	−0.015	0.001	−12.847∗∗	−0.017, −0.013
*R* ^2^	0.589				0.754			
*F*	145.394				282.936			

^∗∗^represents *p* < 0.01.

For the Low perceived intergenerational communication group, as social alienation increases, psychological resilience also increases significantly (*β* = 0.59, *p* < 0.001). This indicates a positive relationship between social alienation and psychological resilience for individuals with low perceived care-related autonomy.

In the Medium perceived intergenerational communication group, as social alienation increases, psychological resilience decreases slightly (*β* = −0.71, *p* < 0.001). This suggests a slight negative relationship between social alienation and psychological resilience for individuals with medium perceived intergenerational communication.

**Figure 3:**
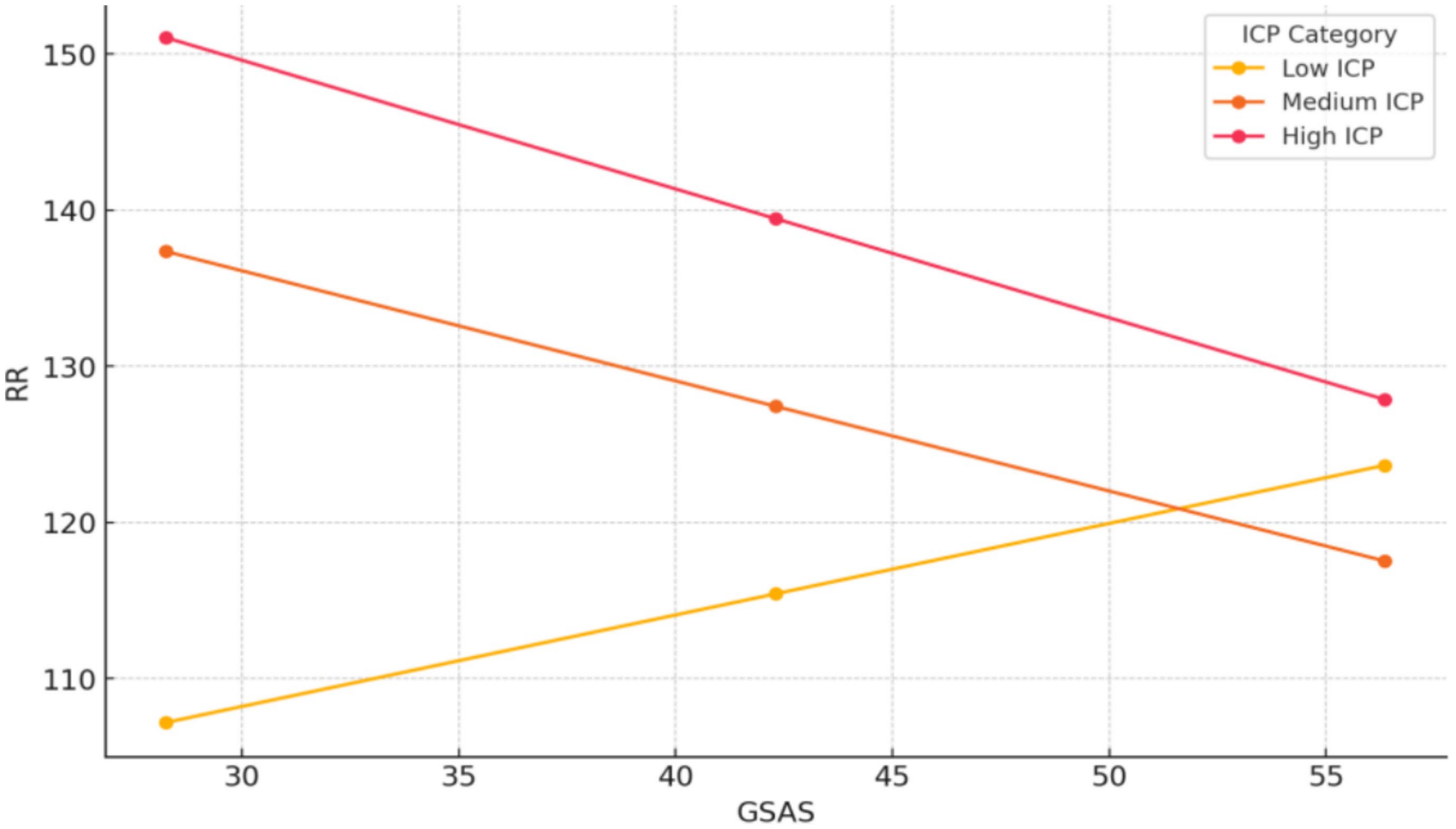
The moderating role of perceived intergenerational communication between social alienation and psychological resilience. ICP, perceived intergenerational communication; GSAS, social alienation; RR, psychological resilience.

For the High perceived intergenerational communication group, as social alienation increases, psychological resilience decreases significantly (*β* = −0.82, *p* < 0.001). This indicates a strong negative relationship between social alienation and psychological resilience for individuals with high perceived intergenerational communication (Figure 3).

**Figure 4:**
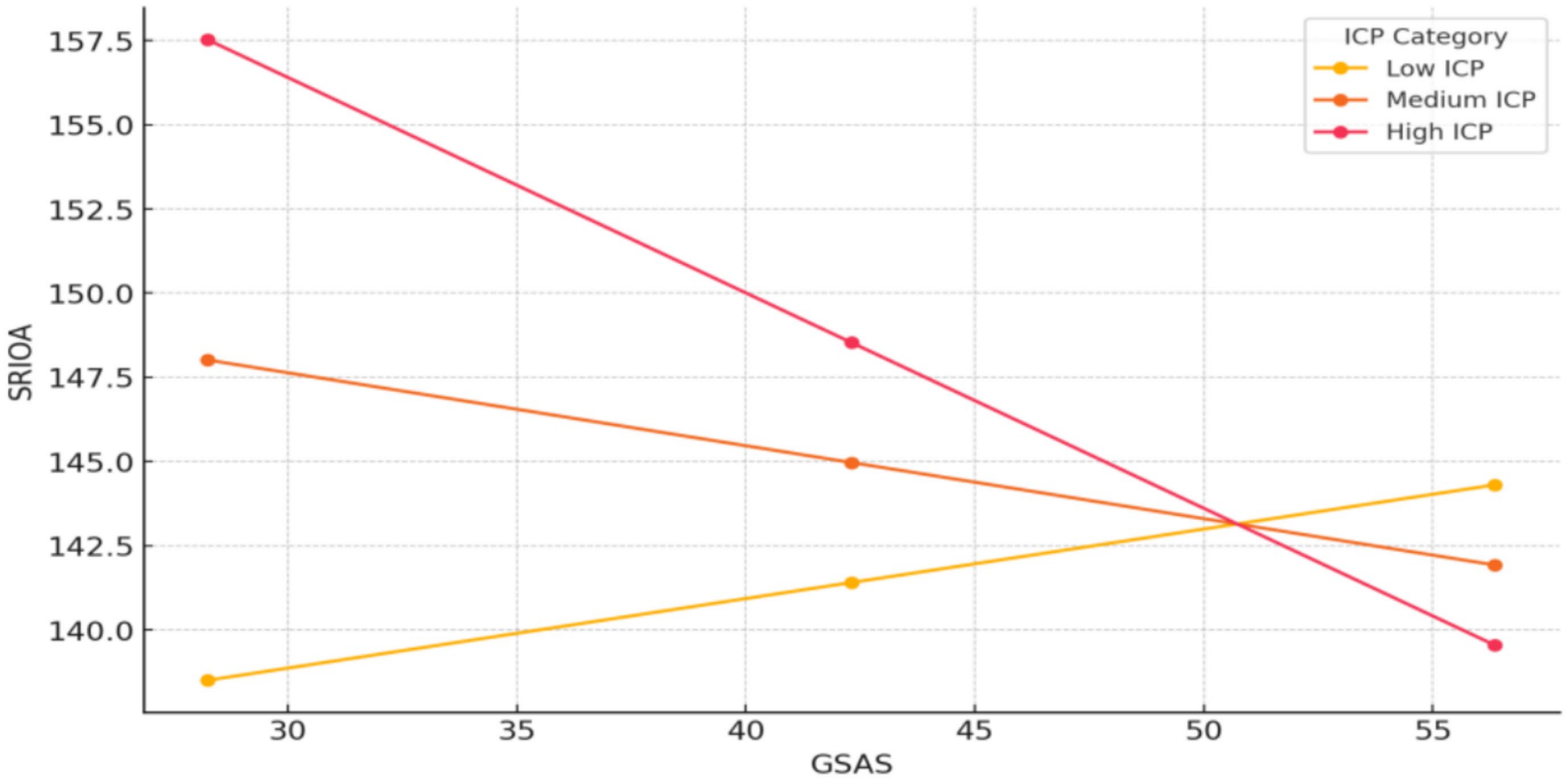
The moderating role of perceived intergenerational communication between social alienation and perceived care-related autonomy. ICP, perceived intergenerational communication; GSAS, social alienation; SRIOA, perceived care-related autonomy.

For the Low perceived intergenerational communication group, as social alienation increases, perceived care-related autonomy also increases (*β* = 0.21, *p* < 0.001). This indicates a positive relationship between social alienation and perceived care-related autonomy for individuals with low perceived intergenerational communication. In the Medium perceived intergenerational communication group, as social alienation increases, perceived care-related autonomy decreases (*β* = −0.22, *p* < 0.001). This suggests a negative relationship between social alienation and perceived care-related autonomy for individuals with medium perceived intergenerational communication. For the High perceived intergenerational communication group, as social alienation increases, perceived care-related autonomy decreases significantly (*β* = −0.64, *p* < 0.001). This indicates a strong negative relationship between social alienation and perceived care-related autonomy for individuals with high perceived intergenerational communication (Figure 4).

These findings collectively highlight the complex interplay between social alienation, psychological resilience, and perceived intergenerational communication in shaping perceived care-related autonomy among rural older adults’ populations. The moderating role of perceived intergenerational communication emerges as a pivotal factor in both direct and mediated relationships within this context.

## 4 Discussion

This cross-sectional study examined associations among social alienation (conceptually distinct from but overlapping with social isolation, emphasizing feelings of disconnection, powerlessness, and reduced social integration), psychological resilience (the capacity for psychological adaptation and recovery in adversity, closely tied to self-efficacy, control beliefs, and adaptive coping), perceived intergenerational communication (a measure of social connectedness through family ties, encompassing emotional support and interaction), and perceived care-related autonomy (perceived care-related autonomy; self-care/self-reliance abilities enabling independent life and health management) among rural older adults. This marginalized population is underrepresented, with literature gaps in perceived social networks in rural settings (e.g., how alienation erodes connectedness, indirectly impacting resilience-driven autonomy); thus, mediation/moderation were investigated exploratorily alongside bidirectional correlations.

### 4.1 The association partially accounted for by psychological resilience

Bidirectional Pearson correlations revealed significant negative associations between social alienation and perceived care-related autonomy (*r* = −0.533, *p* < 0.01), as well as between social alienation and psychological resilience (*r* = −0.566, *p* < 0.01), with a strong positive association between psychological resilience and perceived care-related autonomy (*r* = 0.818, *p* < 0.01; Table 2). Exploratory mediation analysis (Hayes’ PROCESS Model 4, controlling demographics) showed psychological resilience partially accounted for the social alienation-perceived care-related autonomy association [indirect *β* = −0.597, SE = 0.041, 95% CI = (−0.675, −0.519); direct *β* = −0.161, 21.24% of total effect; Tables 3, 4].

The overall mean perceived care-related autonomy score of 145.92 (SD = 21.72) indicates moderate levels relative to the scale’s 0–200 range, comparable to or slightly higher than reported in urban or socioeconomically advantaged older adult samples (e.g., means of 130–145 in studies of self-care autonomy (33, 34)), but univariate analyses showed lower scores associated with objective care needs such as multiple chronic diseases (e.g., 137.70 for ≥3 vs. 152.38 for none; Table 1). This suggests perceived autonomy inversely correlates with objective care demands, potentially mediated by diminished self-efficacy in higher-need groups—a pattern more pronounced in rural settings lacking urban resources.

Psychological resilience and perceived care-related autonomy are clinically crucial constructs: resilience enables older adults to maintain control beliefs and independence amid adversity, while perceived care-related autonomy directly supports daily health management and quality of life, particularly vital for rural older adults facing physical/social challenges (35, 36). Empirical patterns substantiate these associations (11, 37), with resilience correlating positively with self-care abilities, life satisfaction, physical health, and social engagement in rural populations. When rural older adults experience social alienation (e.g., loneliness, limited intergenerational ties), higher resilience relates to positive coping strategies like optimism, hope, and self-efficacy, which in turn correlate with greater perceived care-related autonomy.

#### 4.1.1 Clinical and practical implications

These exploratory patterns suggest potential value in interventions relating to resilience, such as psychological support (paramount via counseling to develop positive outlooks and cope with challenges), strengthening social networks (community activities to boost interaction and mindset adjustment amid alienation), and promoting intergenerational communication (younger generations providing concern/companionship for mutual trust and optimism). Families/society addressing psychological needs may support resilience, relating to enhanced perceived care-related autonomy and happiness.

### 4.2 The moderating associations of perceived intergenerational communication

Correlations indicated negative associations between perceived intergenerational communication and social alienation (*r* = −0.160, *p* < 0.01), positive with resilience (*r* = 0.570, p < 0.01), and perceived care-related autonomy (*r* = 0.589, *p* < 0.01). Exploratory moderated mediation (PROCESS Model 8) showed perceived intergenerational communication moderated the social alienation-resilience association [interaction *β* = −0.004, 95% CI = (−0.007, −0.001)] and resilience-perceived care-related autonomy link [*β* = −0.015, 95% CI = (−0.017, −0.013); Table 5, Figure 2], with stronger communication amplifying negative slopes in high groups (Figures 3, 4).

The scale was treated uni-dimensionally due to strong internal consistency, effectively capturing overall connectedness; however, future research could examine sub-dimensions (e.g., emotional support correlating with resilience, daily interaction with perceived care-related autonomy) for nuanced insights.

Within intergenerational relationship theory, these patterns relate to how communication fosters understanding/support, correlating with well-being and reduced disconnection—especially relevant rurally amid family migration [intergenerational theory]. Timonen et al. (33), [Intergenerational Solidarity Theory] posit relationship quality associates with older adults’ well-being; positive perception correlates with emotional connections, weakening alienation’s impact on resilience (34). Communication also relates to resource sharing (e.g., youth aiding medical access), buffering alienation-perceived care-related autonomy in empty-nesters (38). Kirk et al. (39) found positive ties associate with engagement/health management, contrasting weaker rural vs. urban connectedness.

#### 4.2.1 Clinical implications for rural vs. urban

Rural patterns highlight unique vulnerabilities absent in advantaged urban samples (stronger family networks); interventions like family respect/listening (40), caregiver training (41), community gardens/cultural events/workshops (42), volunteer opportunities (43), accessible mental health/policies (44), age-friendly spaces/awareness campaigns (45, 46) may enhance connectedness, resilience, and perceived care-related autonomy.

In summary, enhancing intergenerational patterns relates to addressing rural aging challenges through collaborative efforts.

### 4.3 The integration of theory and practice

These findings enrich resilience/alienation theories by linking low connectedness (alienation/communication) to reduced self-efficacy/independence (resilience/perceived care-related autonomy), filling rural gaps. The rural sample provides strengths for marginalized insights, differing from urban/advantaged (e.g., higher baseline resilience (34)). Practice: policymakers/social workers prioritize resilience/communication strategies for quality-of-life gains; clinical applications from associational patterns warrant longitudinal validation.

## 5 Research limitations and future directions

Continuous data collection during COVID-19 blended phases, potentially influencing mindsets via stressors and limiting generalizability. Social Alienation Scale subscale reliability varied (*α* = 0.614–0.772; low powerlessness), recommending higher-reliability alternatives. No national database comparisons possible; rural focus offers novel marginalized insights but needs urban/advantaged replication. Sample/regional specificity noted; broader factors (e.g., objective autonomy) unexplored.

### 5.1 Future directions

Longitudinal/experimental designs to assess causality/replication; sub-dimension analyses; parsimonious models with national data; comparative urban/rural studies.

## 6 Conclusion

The exploratory analysis revealed bidirectional associations, indicating that social alienation is negatively related to psychological capital. This relationship was found to be partly mediated by resilience and further moderated by the quality of intergenerational communication. These patterns help to clarify important theoretical links, particularly between social connectedness and self-efficacy. The findings offer valuable insights for clinical practice and social policy aimed at enhancing the health and well-being of rural older adults. Future studies employing more advanced research designs are needed to confirm and extend these preliminary observations.

## Data Availability

The raw data supporting the conclusions of this article will be made available by the authors, without undue reservation.
